# Retracted: Consensus Guidelines for CSF and Blood Biobanking for CNS Biomarker Studies

**DOI:** 10.1155/2016/8304273

**Published:** 2016-07-25

**Authors:** Multiple Sclerosis International

*Multiple Sclerosis International *has retracted the article titled “Consensus Guidelines for CSF and Blood Biobanking for CNS Biomarker Studies” [1] for duplicate publication, due to a misunderstanding. The article was previously published as C. E. Teunissen, A. Petzold, J. L. Bennett, F. S. Berven, L. Brundin, M. Comabella, D. Franciotta, J. L. Frederiksen, J. O. Fleming, R. Furlan, R. Q. Hintzen, MD, S. G. Hughes, M. H. Johnson, E. Krasulova, J. Kuhle, M. C. Magnone, C. Rajda, K. Rejdak, H. K. Schmidt, V. van Pesch, E. Waubant, C. Wolf, G. Giovannoni, B. Hemmer, H. Tumani, F. Deisenhammer: A consensus protocol for the standardization of cerebrospinal fluid collection and biobanking.* Neurology* December 1, 2009; 73(22): 1914–1922. doi: 10.1212/WNL.0b013e3181c47cc2 [2]. Publication was approved by* Multiple Sclerosis International* and the authors, but an arrangement with the American Academy of Neurology and its publisher, Wolters Kluwer, to republish these guidelines did not include permission to publish in this journal.
